# The influence of childhood socioeconomic status on Chinese female university students’ fertility intentions: the chain mediating effect of parental emotional warmth and subjective well-being

**DOI:** 10.3389/fpsyg.2025.1620780

**Published:** 2025-08-20

**Authors:** Junli Hua, Leiyan Mao, Huiyun Liao, Yihui Li, Haoyao Zhang, Wanqi Liu, Hong Tang

**Affiliations:** Department of Psychology, Gannan Medical University, Ganzhou, Jiangxi, China

**Keywords:** childhood socioeconomic status, parental emotional warmth, subjective well-being, fertility intentions, Chinese female university students

## Abstract

**Objective:**

This study examines the impact of childhood socioeconomic status on Chinese female university students’ fertility intentions and the mechanisms by which parental emotional warmth and subjective well-being play a role.

**Method:**

A total of 590 female university students at Gannan Medical University completed questionnaires. The childhood socioeconomic status scale, the simplified parenting styles questionnaire, the index of well-being, and fertility intention scale were used to measure childhood socioeconomic status, parental emotional warmth, subjective well-being, and Chinese female university students’ fertility intentions.

**Results:**

(1) Childhood socioeconomic status, parental emotional warmth, subjective well-being, and fertility intentions among Chinese female university students were significantly positively correlated; (2) Childhood socioeconomic status significantly and positively predicted the fertility intentions of Chinese female university students; (3) Fathers’ emotional warmth and subjective well-being played separate mediating roles in the influence of childhood socioeconomic status on the fertility intentions of Chinese female university students; and (4) Parental emotional warmth and subjective well-being were chain mediators of the effect of childhood socioeconomic status on Chinese female university students’ fertility intentions.

**Conclusion:**

The results indicate that childhood socioeconomic status influences the fertility intentions of Chinese female university students both directly and indirectly. The indirect effect occurs through a chain mediation process involving parental emotional warmth and subjective well-being.

## 1 Introduction

Since the 1990s, the global birth rate has declined, and this phenomenon has become increasingly significant in China. In 2023, China’s birth rate fell to 0.639%, and its natural population growth rate reached an unprecedented negative value of −0.148% for the first time in decades (Statistics Bureau China National, 2024—National Bureau of Statistics of China, 2025). Current population projections show that by 2035, the elderly population will account for more than 24.2% (Qiao, 2024). These pose tremendous challenges to China’s socio-economic development. In terms of fertility rate, China’s one-child fertility rate was 0.73 in 2010 and dropped to 0.52 by 2021, reflecting the generally low fertility intentions among young people of childbearing age (Zhou et al., 2021). Fertility intentions refer to an individual’s subjective considerations regarding having children. As a predictive indicator, they help assess actual childbearing behavior and predict demographic shifts, while also reflecting, to some extent, the potential for increasing fertility levels within a population (Huang and Xing, 2022). To enhance the fertility intentions of individuals of childbearing age, on October 28, 2024, the General Office of the State Council released “Several Measures to Accelerate the Improvement of the Fertility Support Policy System and Promote the Construction of a Fertility-Friendly Society” (Official Gazette of the State Council of the People's Republic of China, 2024). It is worth noting that China’s higher education sector has experienced steady expansion, as university student populations have demonstrated consistent annual growth. These young people have varying characteristics: family-oriented and individual-oriented, idealist and realist, and traditional and modern values, presenting a pluralistic image of youth in the context of compressed modernity (Yu and Zhang, 2024). As potential parents of the next generation, university students, especially the females, not only represent reproductive potential, but also provide insight into China’s future marriage and fertility patterns. Therefore, investigating their fertility intentions is crucial for predicting demographic trends and refining policies aimed at curbing the national fertility decline. In summary, this study aims to explore the factors (and their potential mechanisms) that influence the fertility intentions among female university students in China.

The ecological systems theory holds that an individual’s development is molded by a sequence of nested and interacting environmental systems. Throughout the developmental process, the influences of family environment and life circumstances exhibit long-term continuity (Bronfenbrenner, 1986). These early environmental factors, particularly the childhood environment, are believed to significantly influence various aspects of an individual’s life course, including their mating strategies and reproductive outcomes (Dinh et al., 2022). Furthermore, an individual’s childhood socioeconomic status can significantly influence his or her psychology and behavior in adulthood, which is a core determinant of one’s childhood environment (Jin, 2022). Childhood socioeconomic status refers to a comprehensive consideration of an individual’s family socioeconomic status during childhood, including family income, education level, occupational status, and many other elements. This concept indicates the extent of resources available to individuals in their early life environment (Bradley and Corwyn, 2002; Ellis et al., 2009; Guo and Harris, 2000). Empirical studies have demonstrated that childhood socioeconomic status influences individual fertility intentions through the formation of life history strategies. Specifically, it has been established that a higher socioeconomic status in childhood leads to slow, future-oriented life-history strategies, which demonstrate a positive correlation with elevated fertility intentions (Sun and Wang, 2023). Moreover, studies have indicated that women from higher socioeconomic backgrounds, particularly those raised in economically advantaged families, exhibit more pronounced fertility intentions (Lan, 2021). Building on these findings, we posited the following hypothesis:

*H*1: Childhood socioeconomic status positively predicts the fertility intentions of Chinese female university students.

### 1.1 The mediating role of parental emotional warmth

Within the purview of the family systems theory, the family is recognized as a cohesive unit, wherein the reciprocal interactions among family members frequently and profoundly influences the psychological and behavioral outcomes of other family constituents (Cox and Paley, 1997). As individuals progress through adolescence and approach marriageable and childbearing age, they continue to be subtly influenced by their parents over an extended period, often spanning two decades or more. Parenting style encompasses the aggregate of concepts, attitudes, emotions, and all other verbal and behavioral expressions that parents exhibit while rearing their offspring (Darling and Steinberg, 1993). Drawing upon the research of Swiss scholars (Perris et al., 1980), domestic researchers Jiang et al. have delineated three distinct parenting styles: rejection, emotional warmth, and overprotection (Jiang et al., 2010). Rejection is characterized by hostile, severe, and punitive parenting behaviors; emotional warmth pertains to the affective acceptance and support parents provide for their children; and overprotection involves excessive interference, stringent management, and control over children’s lives. Compared to rejection or overprotection, emotional warmth is more frequently found to have a significantly positive impact on children’s psychological and social development. For example, it can enhance children’s sense of social responsibility and social emotional skills (Shi, 2018; Yao and Chen, 2022). These traits are closely linked to positive fertility intentions (Shi, 2018; Zhou, 2023). Moreover, empirical studies have shown that a emotional warmth can positively predict the fertility intentions of university students. Specifically, this parenting style is conducive to the formation of a sense of social responsibility among university students (Shi, 2018), and a higher sense of social responsibility can further strengthen an individual’s fertility intentions (Luo et al., 2018). Meanwhile, some scholars have pointed out that parental care has a positive impact on the fertility intentions of young people (Xu, 2019). It is apparent, then, that a parenting style characterized by emotional warmth might be a significant predictor of fertility intentions among Chinese female university students. Additionally, socioeconomic status in childhood might also be positively associated with parental emotional warmth. According to the family stress and family investment models, parents in low socioeconomic status families might experience higher levels of psychological distress, which can predict adverse parenting behavior (Conger and Donnellan, 2007). In contrast, studies have suggested that parents in families with higher socioeconomic status are more likely to exhibit emotional warmth in their parenting (Zhang and Wang, 2020). Based on these considerations, a second hypothesis was posited.

*H*2: Parental emotional warmth has a significant mediating effect between childhood socioeconomic status and the fertility intentions among Chinese female university students, that is, a higher childhood socioeconomic status is associated with a greater level of parental emotional warmth, which in turn increases Chinese female university students’ fertility intentions.

### 1.2 The mediating role of subjective well-being

The theory of planned behavior delineates the influence on human fertility behavior as being contingent upon three pivotal factors: subjective attitudes toward fertility behavior, subjective norms pertaining to fertility behavior, and potential behavioral control regarding fertility. Individuals who manifest positive attitudes toward fertility behavior and expect to derive well-being from it are inclined to exhibit heightened fertility intentions, which are ultimately reflected in a greater number of actual progeny (Ajzen and Klobas, 2013). In other words, subjective well-being might have an influence on an individual’s fertility intentions. Subjective well-being refers to an individual’s comprehensive evaluation of their emotional experiences and overall life satisfaction (Diener, 1984). Extant literature, including studies conducted by both international and domestic scholars, has consistently demonstrated that the enhancement of subjective well-being is significantly associated with an escalation in the fertility intentions among the youth (Billari, 2009; Perelli-Harris, 2006; Yang and Xie, 2022; Zhang, 2021). Meanwhile, an individual’s socioeconomic status during childhood is also connected with subjective well-being. Some researchers have suggested that life history strategies exist on a continuum, ranging from “slow” to “fast,” with individuals from lower childhood socioeconomic backgrounds being more likely to adopt a faster life history strategy (Wang et al., 2017). Those with a predisposition toward a faster life history strategy tend to be impulsive, lack long-term planning, and prefer opportunistic behavior. They also exhibit lower levels of delayed gratification, cooperation, and rule conformity (Guan and Zhou, 2016; Hengartner, 2017), all of which are negatively correlated with subjective well-being. Conversely, individuals with a slow approach to life history tend to have higher subjective well-being. This hypothesis is supported by empirical evidence. A greater predisposition toward a quick approach to life history correlates with less subjective well-being, while a greater predisposition toward a slow approach to life history correlates with greater subjective well-being (Figueredo et al., 2007; Zhang et al., 2021). Based on these considerations, a third hypothesis was posited.

*H*3: Subjective well-being plays a critical mediating role in the pathway connecting childhood socioeconomic status and Chinese female university students’ fertility intentions. That is, a higher childhood socioeconomic status is associated with higher subjective well-being, which in turn increases Chinese female university students’ fertility intentions.

### 1.3 Chain mediation of parental emotional warmth and subjective well-being

In summary, both subjective well-being and parental emotional warmth could be the mediator variables of childhood socioeconomic status and Chinese female university students’ fertility intentions, and they also correlate with each other. According to the biosocial cognitive theoretical model, subjective well-being is affected by both external factors and intra-individual factors. External factors include negative life events and parenting styles, while intra-individual factors encompass self-esteem, personality traits, and sense of self-control (Ding and Wang, 2004; Lyons et al., 2013). The attachment theory further explains that neglectful parenting leads to the development of negative internal working patterns and insecure attachment patterns, which reduce an individual’s subjective well-being in adulthood. Positive parenting styles, on the other hand, are conducive for developing a secure attachment style, which enhances an individual’s subjective well-being (Yang et al., 2008). The above view is also confirmed by some studies that show that parental emotional warmth can significantly enhance university students’ subjective well-being, while negative parenting styles are not conducive for subjective well-being (Wei, 2024; Xu, 2018). Therefore, based on the aforementioned content, this study proposes a fourth hypothesis:

*H*4: Parental emotional warmth and subjective well-being exhibit a sequential mediation effect between childhood socioeconomic status and Chinese female university students’ fertility intentions, that is, childhood socioeconomic status is posited to enhance fertility intentions among female university students by sequentially increasing parental warmth and subjective well-being.

**Figure 1:**
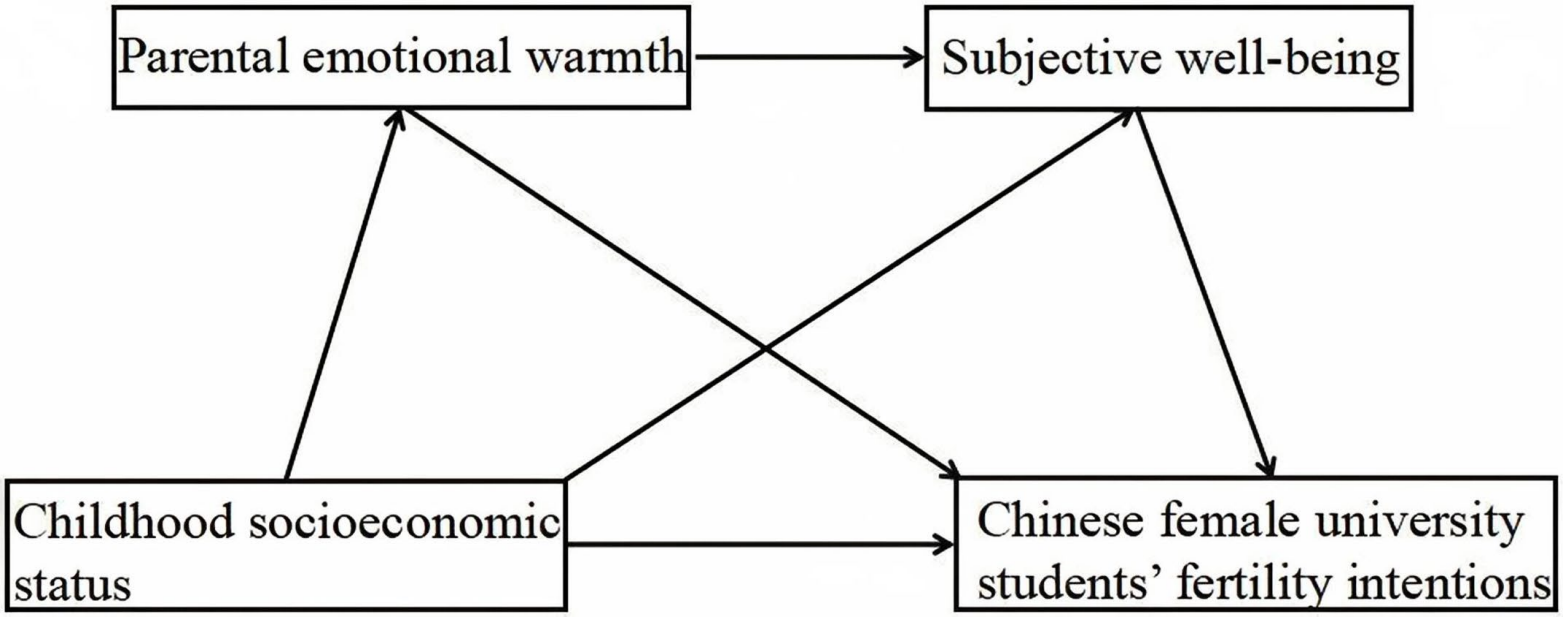
Theoretical framework of the chain mediation model.

In conclusion, the purpose of this study is to investigate the relationship between childhood socioeconomic status, parental emotional warmth, subjective well-being, and fertility intentions of Chinese female university students to elucidate the internal mechanism of the influence of childhood socioeconomic status on the fertility intentions of Chinese female university students. Figure 1 shows the chain mediation model.

## 2 Materials and methods

### 2.1 Participants and procedures

This study utilized G*Power software to conduct *a priori* power analysis. The power analysis determined the minimum required sample size by considering the model component with the most predictors (Memon et al., 2020). By setting the Effect size f^2^ = 0.15, *α* err prob. = 0.05, Power (1-βerr prob) = 0.80, we calculated that the required sample size is 85.

This study recruited participants from female university students at Gannan Medical University using a convenience sampling method. The screening process excluded individuals with intellectual disabilities. After implementing strict quality control measures, a total of 590 valid questionnaires were retained. The average age of these Chinese female university students was 18.93 years old. The demographic composition included 186 first-year students (31.53%), 263 s-year students (44.58%), and 141 third-year students (23.90%).

The study procedures were carried out in accordance with the Declaration of Helsinki. The Institutional Review Board of the Ganan Medical University approved our study protocol (ethics code: gzsyy2025072). We contacted the participants. After a full explanation of the study, all the participants provided written informed consent for participating.

### 2.2 Methodology

#### 2.2.1 Childhood socioeconomic status scale

We employed the Chinese version of the childhood socioeconomic status scale, which was revised by Chinese scholars (Wang et al., 2017). The scale was retrospectively and indirectly measured through adults’ subjective self-assessment. It has four items, each rated on a 7-point Likert scale (where 1 = “not at all consistent” and 7 = “fully consistent”). Higher scores signify a higher childhood socioeconomic status of the Chinese female university students. In this study, the scale showed strong internal consistency, with a Cronbach’s alpha of 0.90. One of the items on the scale is: “Compared to other kids in school, I feel like I’m relatively rich.”

#### 2.2.2 Simplified parenting styles questionnaire

The emotional warmth dimension of the Chinese version of the simplified parenting styles questionnaire (S-EMBU-C) revised by Jiang et al. (2010), served to evaluate parental emotional warmth characteristics in the Chinese female university students’ caregivers. In this study, it was divided into two scales, father’s version and mother’s version, each with 7 questions, using a 4-point Likert scale (1 = never, 4 = always), with higher scores indicating that the respondent’s father or mother adopted such parenting styles more frequently. The internal consistency coefficients for the two scales in this study were 0.84 and 0.86, respectively. The scale uses a self-report format, meaning that the Chinese female university students evaluate their parents’ parenting styles based on their memories. One of the items on the scale is: “I feel a sense of warmth, thoughtfulness, and closeness with my father.”

#### 2.2.3 Index of well-being

We employed the index of well-being (IWB), which was constructed by Campbell et al. (1976). The scale was measured to evaluate a person’s current level of subjective well-being. It is composed of two components: an index of general affect (including eight items) and an index of life satisfaction (with a single item). For scoring, the scores of the first eight items are multiplied by 1 and the score of the last item by 1.1. All nine items are rated on a 7-point Likert scale, with the scores ranging from 2.1 to 14.7. Higher scores represent a higher level of subjective well-being. In this study, the scale showed strong internal consistency, with a Cronbach’s alpha of 0.93. One of the items on the scale is: “1 means ‘bored’ and 7 means ‘interesting’. Please choose a number between 1 and 7 that best describes your current state based on how you really feel.”

#### 2.2.4 Fertility intention scale

We used the fertility intention scale developed by Zheng (2014). The scale was used to assess the fertility intention of the Chinese female university students. It consists of two items: “the degree of fondness for children” (1 = not at all fond of, 5 = very fond of) and “the intensity of fertility intention” (1 = Not at all strong, 5 = Extremely strong), measured using a 5-point Likert scale. A higher score indicates a stronger intention to have children among the respondent. In this study, the scale showed strong internal consistency, with a Cronbach’s alpha of 0.81.

### 2.3 Statistics

The statistical analysis of this study was conducted using SPSS 23.0 and Mplus 8.3. SPSS 23.0 was used for descriptive statistics, correlation analysis, and Little’s MCAR test for missing data, which indicated missing completely at random (*p* > 0.05). Subsequently, Mplus 8.3 was employed to handle the data via Full Information Maximum Likelihood (FIML). Finally, the SEM in Mplus 8.3 was applied to examine the mediating role of parental emotional warmth and subjective well-being between childhood socioeconomic status and Chinese female university students’ fertility intentions.

## 3 Results

### 3.1 Common method bias test

As the research data were exclusively collected through self-reported questionnaires from Chinese female undergraduates, potential common method variance (CMV) could influence the outcomes. To assess this methodological limitation, an unrotated Harmans’ single-factor test was conducted. The exploratory factor analysis identified five components with eigenvalues above the threshold of 1. Notably, the primary component accounted for 37.815% of the total variance – below the critical 40% benchmark–which implies that CMV does not substantially compromise the validity of the findings.

### 3.2 Descriptive statistics and correlation analysis

Table 1 shows the descriptive statistics and correlation analysis results for each variable. The study findings suggest that childhood socioeconomic status, fathers’ and mothers’ emotional warmth, subjective well-being, and Chinese female university students’ fertility intentions show significant positive correlations with each other.

**Table 1:** Descriptive statistics and correlation analysis results for each variable.

Variables	M	SD	1	2	3	4	5
1. Childhood socioeconomic status	3.505	1.482	1				
2. Fathers’ emotional warmth	2.580	0.705	0.404**	1			
3. Mothers’ emotional warmth	2.730	0.746	0.292**	0.617**	1		
4. Subjective well-being	4.503	1.244	0.516**	0.619**	0.511**	1	
5. Chinese female university students’ fertility intentions	2.789	1.261	0.429**	0.378**	0.287**	0.421**	1

**p < 0.01.

### 3.3 Chain mediation effect testing

**Figure 2:**
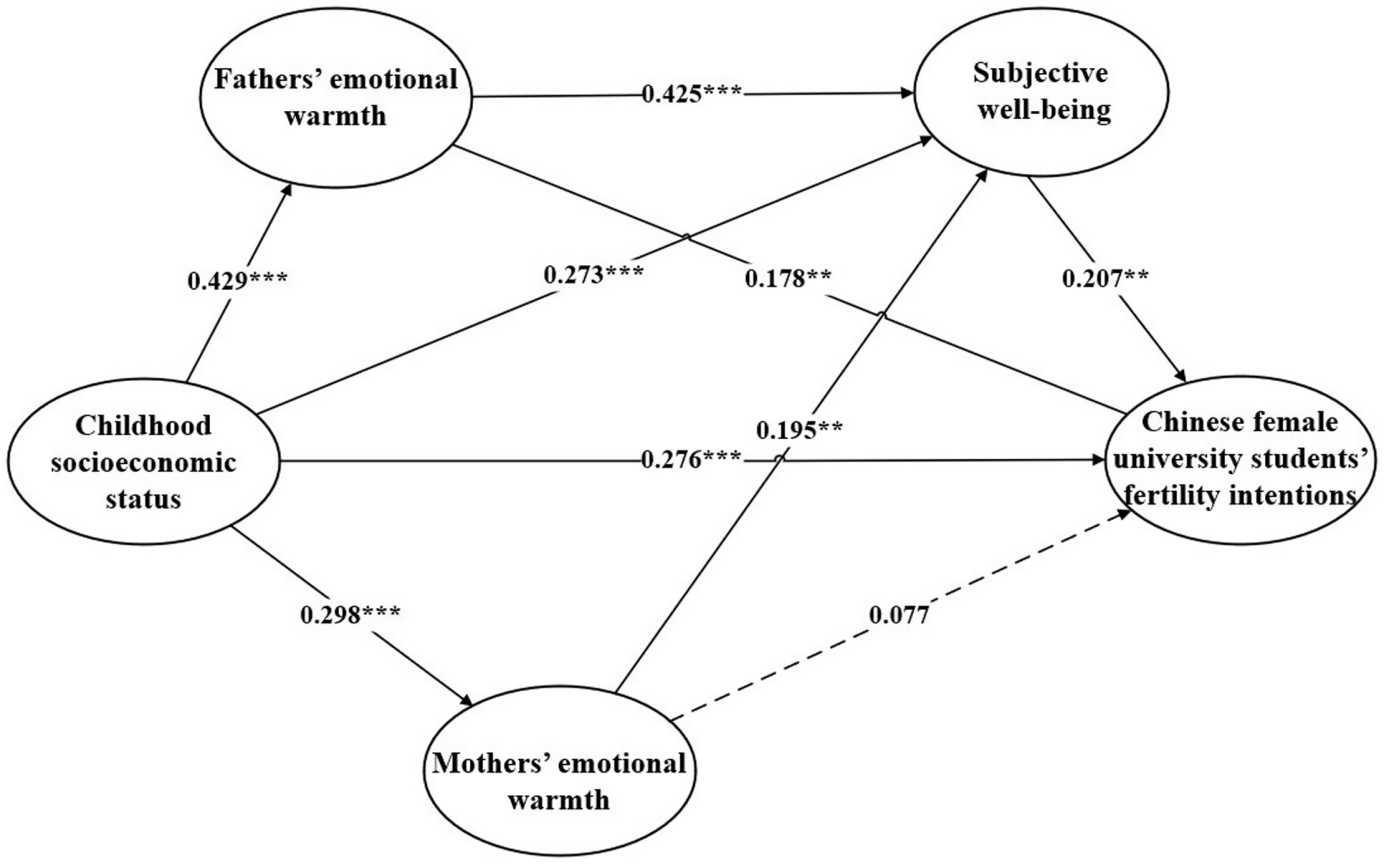
Chain mediation model of Childhood socioeconomic status and Chinese female university students’ fertility intentions between Fathers’ emotional warmth, Mothers’ emotional warmth and Subjective well-being. ***p < 0.01, **p < 0.05.

This study employed Mplus 8.3 to test the chain mediation effect of parental emotional warmth and subjective well-being on the relationship between childhood socioeconomic status and the fertility intentions of Chinese female university students, using the SEM method (Cheung, 2007). The model showed a good fit (x^2^/df = 2.885, CFI = 0.913, TLI = 0.905, RMSEA = 0.057, SRMR = 0.040). Firstly, the direct predictive effect of childhood socioeconomic status on the fertility intentions of Chinese female university students was examined. The results indicated that childhood socioeconomic status significantly positively predicted the fertility intentions of Chinese female university students (*β* = 0.276, *t* = 5.033, *p* < 0.01). Secondly, after including the mediating variables in the model, it was found that childhood socioeconomic status significantly positively predicted fathers’ emotional warmth (*β* = 0.429, *t* = 9.981, *p* < 0.01), mothers’ emotional warmth (*β* = 0.298, *t* = 6.371, *p* < 0.05), and subjective well-being (*β* = 0.273, *t* = 7.140, *p* < 0.01). Fathers’ emotional warmth positively predicted subjective well-being (*β* = 0.425, *t* = 6.538, *p* < 0.01) and the fertility intentions of Chinese female university students (*β* = 0.178, *t* = 2.046, *p* < 0.05). Mothers’ emotional warmth positively predicted subjective well-being (*β* = 0.195, *t* = 3.115, *p* < 0.05), but its positive predictive effect on the fertility intentions of Chinese female university students was not significant (*β* = 0.077, *t* = 0.327, *p* > 0.05). Subjective well-being also positively predicted the fertility intentions of Chinese female university students (*β* = 0.207, *t* = 2.979, *p* < 0.05). The predictive relationships between the variables are presented in Table 2 and Figure 2.

**Table 2:** Regression analysis of variable relationships in the mediation model.

Regression equation variable		Overall fit index of the equation	Significance of regression coefficients	
Outcome variable	Predictor variable	*R^2^*	*β*	*t*
Fathers’ emotional warmth	Childhood socioeconomic status	0.184	0.429	9.981***
Mothers’ emotional warmth	Childhood socioeconomic status	0.089	0.298	6.371***
Subjective well-being	Childhood socioeconomic status	0.541	0.273	7.140***
	Fathers’ emotional warmth		0.425	6.538***
	Mothers’ emotional warmth		0.195	3.115**
Chinese female university students’ fertility intentions	Childhood socioeconomic status	0.360	0.276	5.033***
	Fathers’ emotional warmth		0.178	2.046**
	Mothers’ emotional warmth		0.077	0.327
	Subjective well-being		0.207	2.979**

***p < 0.01, **p < 0.05.

Finally, the Bootstrap bias correction method was used to test the mediating effect results, with 1,000 repeated samplings to calculate the 95%confidence interval. The mediating effect analysis showed that the direct effect was 0.276, with a 95%CI of [0.168, 0.383], accounting for 57.38%of the total effect. The total indirect effect was 0.205, with a 95%CI of [0.143, 0.268], accounting for 42.62%of the total effect. As the confidence intervals did not include 0,the direct and indirect effects were statistically significant. The mediating effect consisted of five paths: (1) The indirect path via childhood socioeconomic status→fathers’ emotional warmth→Chinese female university students’ fertility intentions had an effect size of 0.077,with a 95%CI of [0.003, 0.150], indicating a significant indirect effect. (2) The indirect path via childhood socioeconomic status→mothers’ emotional warmth→Chinese female university students’fertility intentions had an effect size of 0.023,with a 95%CI of[−0.024, 0.070], indicating a nonsignificant indirect effect. (3) The indirect path via childhood socioeconomic status→subjective well-being→Chinese female university students’ fertility intentions had an effect size of 0.056,with a 95%CI of [0.015, 0.097], indicating a significant indirect effect. (4) The indirect path via childhood socioeconomic status→fathers’emotional warmth→subjective well-being→Chinese female university students’fertility intentions had an effect size of 0.038,with a 95%CI of [0.009, 0.066], indicating a significant indirect effect.(5) The indirect path via childhood socioeconomic status→mothers’ emotional warmth→subjective well-being→Chinese female university students’ fertility intentions had an effect size of 0.012,with a 95%CI of [0.001, 0.023], indicating a significant indirect effect. The results are shown in Table 3.

**Table 3:** Direct, indirect, and total effects of the hypothesized mode.

Model pathways	Effect size	Boot SE	95% confidence interval		Relative mediation effect
			LLCI	ULCI	
Total effect	0.481	0.041	0.401	0.562	
Direct effect	0.276	0.055	0.168	0.383	57.38%
Total indirect effect	0.205	0.032	0.143	0.268	42.62%
Childhood socioeconomic status → fathers’ emotional warmth → Chinese female university students’ fertility intentions	0.077	0.037	0.003	0.150	16.01%
Childhood socioeconomic status → mothers’ emotional warmth → Chinese female university students’ fertility intentions	0.023	0.024	−0.024	0.070	4.78%
Childhood socioeconomic status → subjective well-being → Chinese female university students’ fertility intentions	0.056	0.021	0.015	0.097	11.64%
Childhood socioeconomic status → fathers’ emotional warmth → subjective well-being → Chinese female university students’ fertility intentions	0.038	0.015	0.009	0.066	7.90%
Childhood socioeconomic status → mothers’ emotional warmth → subjective well-being → Chinese female university students’ fertility intentions	0.012	0.006	0.001	0.023	2.49%

## 4 Discussion

The results of the study indicate that childhood socioeconomic status is a significant and positive predictor of fertility intentions among Chinese female university students. This result corroborates Hypothesis 1 and is consistent with the conclusions of previous research (Lan, 2021; Sun and Wang, 2023). Chinese female university students from families with higher childhood socioeconomic status typically benefit from greater access to resources that support their overall development. These resources not only meet basic living needs, but also provide enhanced medical care, educational opportunities, and career advancement prospects. Such advantages contribute to the establishment of a stable economic foundation and a higher quality of life in adulthood, which strengthen their ability to withstand the economic and social costs associated with childbirth and child-rearing. Consequently, their fertility intentions are significantly strengthened. Furthermore, in social stratification research, there is a pronounced emphasis on the robust intergenerational continuities in socioeconomic status. This transmission effect leads to the intergenerational convergence of fertility intentions (Kolk, 2014). Research has indicated that higher parental socioeconomic status tends to be associated with stronger fertility intentions (Shi and Wang, 2024), and this trend is perpetuated through the intergenerational transmission, thereby contributing to an escalation in fertility intentions within subsequent progeny.

In this study, fathers’ emotional warmth significantly and positively predicted Chinese female university students’ fertility intentions and mediated the relationship between childhood socioeconomic status and Chinese female university students’ fertility intentions, whereas mothers’ emotional warmth did not significantly and positively predict Chinese female university students’ fertility intentions, suggesting that Hypothesis 2 was partially valid. This supports the conclusions of Shen et al. (2024) but is inconsistent with the findings (Xu, 2023). Higher childhood socioeconomic status usually means that parents can provide more material and spiritual resources to implement warm parenting styles, which enables parents to be more deeply involved in their children’s growth process and provide the necessary support and care. Moreover, parents in families with higher socioeconomic status often exhibit greater educational aspirations and a heightened sense of responsibility toward their children. This motivates them to adopt positive and warm parenting approaches, ultimately fostering their children’s holistic development (Zhao, 2024). In addition, daughters’ perceptions and experiences of their fathers’ significant strengths contribute to the establishment of emotional attachment and satisfaction with their fathers (Lax, 2007), which makes them use their fathers as a reference standard for mate selection process, and this predisposition ultimately has an impact on their decision-making regarding family formation and fertility intentions. In traditional gender division of labor where men are the breadwinners and women are the homemakers, the family parenting order of “strict father and loving mother” has long been prevalent. In contemporary society, this parenting order still holds a significant proportion (Wang et al., 2018; Zhang et al., 2024). In such families, men play the role of the “strict father,” primarily responsible for the social upbringing of their children, including moral and character education. Women, on the other hand, take on the role of the “loving mother,” in charge of emotional support and the physiological care of daily life. Men dominate in their children’s family education, while women are dependent on men. The two work together through this division of labor to complete the upbringing of their children (Fei, 2021). Since fathers hold the dominant position in the family, their emotional warmth can break the traditional “strict father” image, providing Chinese female university students with a new family atmosphere experience different from the traditional authority. This allows them to feel the harmony and warmth of family relationships and are more like to continue this warm family model, thereby increasing their willingness to have children. The father investment theory (Draper and Harpending, 1982; Ellis, 2004) further points out that childhood father investment has a long-term impact on women’s reproductive strategies. Women are very sensitive to the availability and quality of father investment. If a father is absent or the quality of parenting is low, women will think that men are not reliable in long-term investment in offspring, and thus adjust their reproductive strategies. Based on this, the fathers’ emotional warmth will make Chinese female university students feel high-quality father investment and be very sensitive to this parenting style, which in turn increases their willingness to have children.

In this study, subjective well-being significantly and positively predicted fertility intentions among Chinese female university students and mediated the relationship between childhood socioeconomic status and fertility intentions of Chinese female university students. Hypothesis 3 was verified, which aligns with the results of previous studies. The social comparison theory posits that individuals evaluate their own abilities, perspectives, and life circumstances by comparing themselves to others within their social environment (Festinger, 1954). Therefore, Chinese female university students who had a higher socioeconomic status during their childhood tend to feel superior when making social comparisons with their peers, because they may have had more resources and advantages during their formative years. The positive feelings associated with this superiority can enhance their pleasant psychological experience, thus enhancing subjective well-being. In addition, the mood maintenance hypothesis posits that individuals in a positive mood state tend to adopt risk-averse behaviors to maintain their positive mood. Therefore, individuals with higher subjective well-being may have more children to avoid potential risks in old age (Isen and Patrick, 1983). Meanwhile, based on the perspective of the economist regarding the demand for children (Becker, 1976), children can be used as a durable raw product or durable consumer goods from which to derive economic utility, psychological utility, etc. for a sustained sense of well-being. In summary, Chinese female university students with high subjective well-being may believe that children not only bring sustained well-being, but also provide economic and psychological support, which in turn enhances their fertility intentions.

This study also revealed that parental emotional warmth and subjective well-being play a chain mediating role in the effect of childhood socioeconomic status on fertility intentions of Chinese female university students. Hypothesis 4 was supported, aligning with findings from prior studies in this field (Wei, 2024; Xu, 2023). Chinese female university students who grew up with a warm and positive parenting style were able to feel more attention, love, and support from their parents. This parenting style helps to develop the attributional style for positive events (Xu, 2018), enhances an individual’s self-esteem (Liu, 2022), and motivates them to adopt positive emotion regulation strategies to reduce their experience of negative emotions (Hu et al., 2017). Studies have shown that positive attributional styles, high self-esteem, and adaptive emotion regulation strategies show positive correlations with subjective well-being (Furnham and Cheng, 2000; Goldin et al., 2008; Xu, 2018). Consequently, parental emotional warmth positively affects the subjective well-being of Chinese female university students. Those with higher childhood socioeconomic status are more likely to experience parental emotional warmth. This parenting style can enhance their subjective well-being, which can increase their fertility intentions.

### 4.1 Limitations and future studies

Although this study provides important insights into understanding the factors that influence Chinese female university students’ fertility intentions, it has some limitations.

First, this study focuses on female university students at Gannan Medical University in Jiangxi Province. Jiangxi Province is characterized by a fertility-friendly policy-oriented model, and its fertility level is relatively higher than that of other provinces due to the impact of fertility policies (Xie, 2023). Therefore, the generalizability of the findings of this study may be somewhat limited. Future research could further investigate the differences in fertility intentions between female university students in Jiangxi Province and those in other regions in China, in order to gain a more comprehensive understanding of the geographical differences in fertility intentions. Additionally, the subjects of this study are Chinese female medical university students. Due to their professional background and career planning, medical students tend to delay family plans and desire fewer children (Ren et al., 2023). This characteristic may have affected the representativeness of the study’s results. Thus, future research should include Chinese female university students from other majors (such as liberal arts and engineering) and conduct comparative analyses with Chinese female medical students to more comprehensively reveal the impact of different professional backgrounds on fertility intentions. Existing research has demonstrated that family social capital significantly promotes the fertility intentions of Chinese university students (Xu and Sun, 2025). However, this study did not explore the impact of this factor. Future research could conduct stratified analyses of female university students from different family social capital backgrounds to delve into the mechanisms through which family social capital affects fertility intentions. Additionally, the sample of this study may have included a small proportion of Chinese female university students with specific background characteristics, such as those who experienced being left-behind children or grew up in single-parent families. These groups’ unique experiences may have certain impacts on their fertility intentions. Future research could further focus on these particular groups to more accurately understand the differences in fertility intentions among Chinese female university students with diverse backgrounds.

Secondly, this study explored how parental emotional warmth and subjective well-being mediate the relationship between childhood socioeconomic status and fertility intentions among female university students in China. Although this study provides valuable insights, other variables may also affect the association between childhood socioeconomic status and fertility intentions among Chinese female university students. Future studies are encouraged to investigate this relationship more comprehensively by identifying and examining additional mediating variables that could clarify the connection between childhood socioeconomic status and fertility intentions among Chinese female university students.

Thirdly, this study employed convenience sampling. While this method offers advantages such as ease of implementation and low survey costs, its drawback lies in the arbitrary nature of sample selection, which may fail to represent clearly defined populations. Future research should therefore adopt more rigorous probability sampling methods (such as stratified random sampling based on grade level, major, or class) to enhance the reliability and representativeness of the results.

Finally, in the study, the measurement of childhood socioeconomic status relied on retrospective self-reports from Chinese female university students. This could be subject to recall bias, which may affect the accuracy of the data. Future research could consider incorporating objective indicators such as parents’ educational levels, occupations, and family income records, or information from multiple sources. Additionally, although the Simplified Parenting Styles Questionnaire used in this study has good reliability and validity, it was primarily developed based on Western cultural contexts and may not fully capture the unique connotations and expressions of parental warmth in the Chinese cultural context. Therefore, future research could consider developing or using measurement tools that are more culturally sensitive.

By overcoming these limitations and exploring new research areas, future studies can build on the findings of this study to gain a more refined and comprehensive understanding of how childhood socioeconomic status impacts the fertility intentions of Chinese female university students.

## 5 Conclusion

In conclusion, the study shows that childhood socioeconomic status positively predicts the fertility intentions of Chinese female university students, with fathers’ emotional warmth and subjective well-being acting as independent and chained mediators. This research highlights psychological factors affecting Chinese female university students’ fertility intentions and offers practical suggestions. Policymakers should boost economic support and resources for low-income families to counteract low fertility intentions caused by childhood deprivation. Educational and community organizations can foster warm, supportive parenting via a comprehensive family education system. The government and social groups should also provide social welfare and employment protections to improve young women’s well-being and fertility intentions.

## Data Availability

The original contributions presented in the study are included in the article/Supplementary material, further inquiries can be directed to the corresponding author.
